# Correlation of internet search enquiries, incidence of ophthalmological diseases and corneal, cataract and refractive surgical procedures

**DOI:** 10.1038/s41598-022-26704-y

**Published:** 2022-12-23

**Authors:** Mohamed Ghaly, Diana Jaber, Mario Matthaei, Claus Cursiefen, Alexander Händel, Juliane Köberlein-Neu, Robert Hörster, Sebastian Siebelmann

**Affiliations:** 1 Department of Ophthalmology, University of Cologne, Cologne, Germany; 2 AOB-Augenärzte-Augenkliniken/Artemis, Ballindamm 37, Hamburg, Germany; 3 University of Applied Science HAW, Hamburg, Germany; 4 Bergische Universität Wuppertal, Wuppertal, Germany; 5 Augencentrum Erkelenz, Erkelenz, Germany

**Keywords:** Social behaviour, Computational biology and bioinformatics, Health care, Medical research

## Abstract

Analysis of internet search queries (ISQ) could be useful to study public interest and medical need for corneal, cataract, and refractive surgery. To date, there are preliminary data on seasonal trends in ophthalmic conditions, but only few studies correlate these data with real data from healthcare systems. The aim of this study is to analyze ISQ and correlate it with real healthcare system data. Data were retrieved from the KBV registry of patients who underwent outpatient ophthalmic surgery in Germany from 2017 to 2019 and from Statista GmbH from 2010 to 2020 for corneal refractive surgery. Time Series analysis of ISQ was analyzed from 2004 to 2020 and correlated with healthcare system data using bivariate correlation analysis. ISQ correlated significantly with the incidence of ophthalmic procedures such as corneal transplantations (r = 0.69, *p* < 0.05), cataract- (r = 0.59, *p* < 0.05) and refractive laser surgery (r = 0.83, *p* < 0.05) in Germany. In addition, specific trends were observed with respect to individual surgical procedures. The correlation between search intensities and surgical procedures varied significantly. Thus, interests in surgical procedures can be tracked by observing changes in ISQ over time. These data correlate with real healthcare data and could be used in the future for now-casting or even forecasting.

## Introduction

In 2016, it was estimated that almost half of the world's population used the internet. Amongst the world population the availability of the internet was over 80 per cent in developed regions and just under 40 per cent in developing countries^1^. In Europe, over 75 per cent consider the internet as a good way to learn more about health topics. Six out of ten Europeans go online when looking for health information. 90 percent of them said that the internet has helped them improve their knowledge about health-related topics^2^. In Germany, about 46 percent of respondents said, that they regularly research health topics on the internet^3^.

Google Trends (GT) is the main tool used to study trends and patterns of search engine queries with Google and is a free online tool of the company Alphabet Inc., (Mountain View, California, USA) that is primarily used to query search queries, topics or phrases over a specific time period. These queries, in turn, are presented in charts that reflect the public interest of users and are intended to allow forecasts. The popularity of individual search terms can be analyzed over time and compared in relation to other terms regionally or worldwide. Google Trends draws on this enormous mass of search queries to display social trends in visually appealing diagrams.

So far, the analysis of internet search engine queries (ISQ) has been successfully used with regard to different questions. The analyses range from the seasonality of search queries to the correlation of search engine queries with data from third-party sources, the modelling and visualization of search engine queries, for example, according to geographical distribution, as well as the inventory of queries in real time and the resulting prediction of developments in the future (nowcasting, forecasting)^4^.

In the health sector, there are also numerous studies worldwide that describe the benefits of evaluating search engine queries to determine the spread of infectious diseases such as Ebola,influenza and recently COVID-19^5^. This is particularly due to the fact that even in countries with very low incomes, smartphones are almost ubiquitous^6^. Furthermore, such data sets can also be used to predict the demand for certain surgical interventions. This has been demonstrated, for example, in bariatric surgery^7^.Interestingly, however, it has been shown that the correlation between ISQ and certain diseases or operations is not unconditional, but is in some cases weak or even non-existent^8^.

In the field of ophthalmology, however, there is virtually no data on this to date. So far, for example, it was possible to identify certain epidemics of conjunctivitis worldwide with the help of search engine queries^9^. On the other hand, however, it was shown that the search trend for laser-assisted in situ keratomileusis (LASIK) is decreasing significantly, while the number of procedures performed is increasing strongly in Europe^10^. However, to date in ophthalmology, these search intensities have never been linked or correlated to real healthcare system data**.** This study examines whether the information provided by ISQ generated from Google Trends is correlated with real-life data of procedures of cataract, corneal and refractive surgery in Germany.

## Materials and methods

### Objectives/hypotheses

This study examines the following objectives/hypotheses:

1. It is assumed that certain internet search queries regarding different corneal, cataract and refractive surgery operations follow a time-dependent, characteristic course. This course will be described.
2. In addition, the temporal and geographic pattern of internet searches is assumed to correspond and correlate with the actual occurrence of each surgical procedure. This will be analyzed by performing a correlation analysis, in which real health data will be correlated with the course of internet search queries. It is assumed that certain search terms correlate well with real operation numbers and some less well.

### Data acquisition from the association of statutory health insurance physicians in Germany (Kassenärztliche Bundesvereinigung (KBV))

As the umbrella organization of the individual associations of panel doctors, KBV occupies a key position in the statutory health insurance system in Germany.

As part of a collaboration with the National Association of Statutory Health Insurance Physicians (KBV), three-monthly totals of surgical procedures done in outpatient care according to specific operation and procedures codes—OPS of phacoemulsification (OPS: 31,351/36,351), posterior lamellar keratoplasties (Descemet Membrane Endothelial Keratoplasty; DMEK and DSAEK) (OPS: 31,334), and penetrating keratoplasty (OPS: 31,334) were analyzed for the period from January 2017 to June 2019 in Germany. Data were queried only for the total number of previously mentioned procedures without personal data of the patients. The annual number of LASIK eye surgery was queried via free available data from Statista.de (Statista GmbH, Hamburg, Germany) in the period from 2010 till 2020. The characteristics age, gender or pervious illnesses were not considered in this study. The study adhered to the Declaration of Helsinki. As no patient’s data were used, no approval of the Ethics Committee was necessary. No human participants were involved in this study.

### Google trends

Data was queried via the Google Trends tool (http://google.com/trends). The popularity of individual search terms can be analyzed over time and even compared in relation to other terms regionally or worldwide. The time frame and the region can be changed manually at any time. If the time frame is three months or shorter, the data is displayed on a daily basis. If the time frame is longer than three months, the results are staggered on a weekly basis. GT's graphical data does not convey absolute numbers on the search volume of terms in the context of ISQ. Instead, the numbers in the graphs reflect how many searches were conducted for a particular term relative to the total number of searches on Google over time (Relative Search Volume-RSV). Therefore, a value of 100 is considered the peak popularity of the term, a value of 50 means the term is half as popular, and all numbers in between indicate a percentage of the total peak interest at a given time^11^. In this study RSV of different search terms related to cataract surgery, corneal transplantations and laser refractive surgery were investigated for the period from January 2014 to December 2020 in Germany. Furthermore, for the period from January 2017 to June 2019, a possible correlation with the number of performed surgeries in Germany was analyzed.

### Data analysis

To determine search keywords related to cataract, corneal and refractive surgery for Google Trends queries, we first conducted an electronic search of online and print news articles related to patients interests in Germany. Search keywords related to cataract, corneal and refractive surgery were entered in German language into the Google Trends web interface to generate search interest data in Germany. The data from Google Trends were collected in terms of RSV for specific search terms over the period from 2004 till 2020 in Germany. The RSV of each search terms has been plotted in scatter graph with fitted polynomial trends lines. It has been previously reported that fitted spline polynomial trend lines best fit fluctuating temporal data such as those generated in Google Trends^7,11,12^. Trend analyses were then performed using Microsoft Excel Version 16.57 (Microsoft, Redmond, WA, USA). Final searches in Google Trends were performed on 10. August 2021.

**Figure 1:**
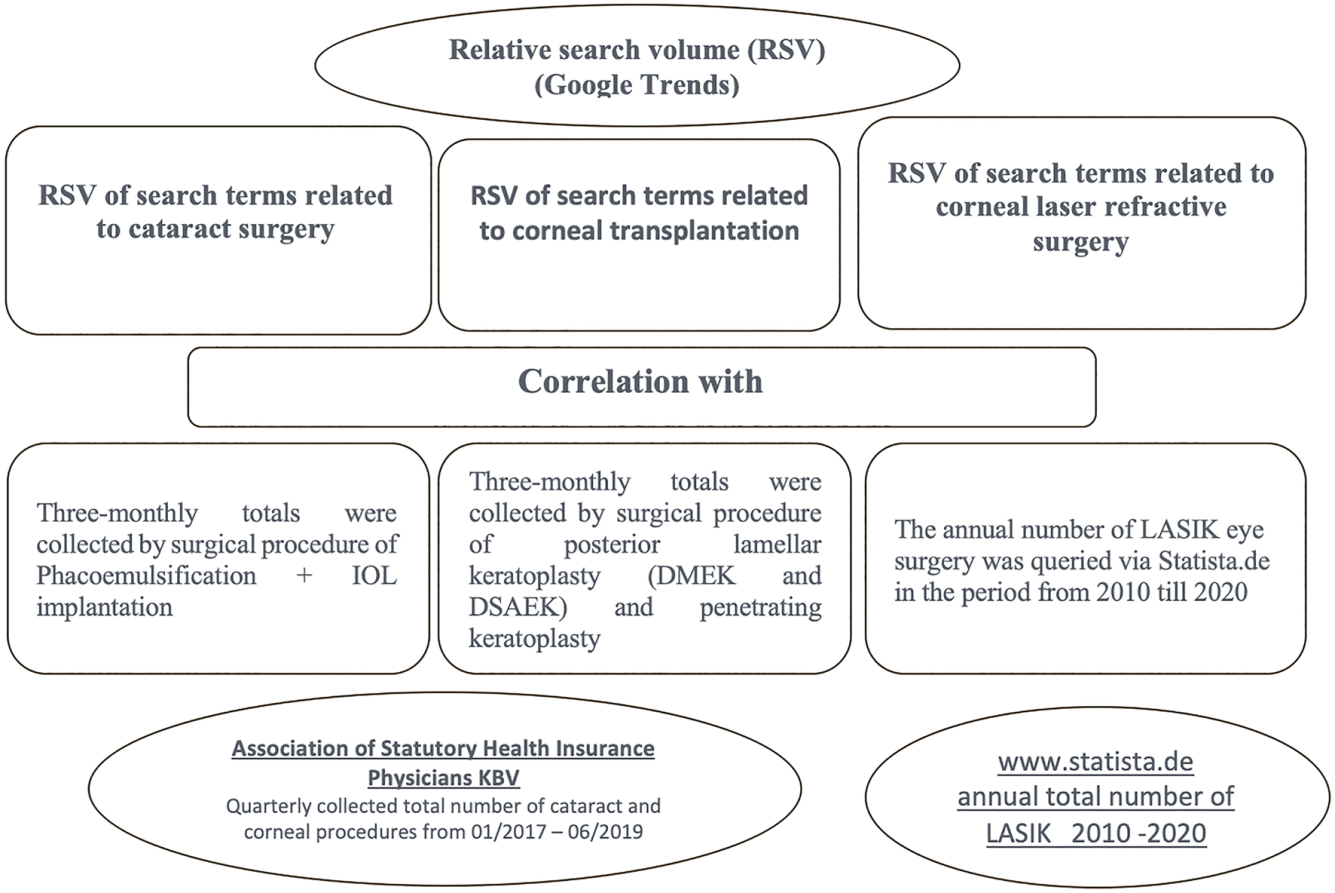
Overview of data acquisition and correlation in this study. Pearson correlation was calculated between the Relative Search Volume (RSV) of surgery specific terms and the number of corresponding procedures done in Germany.

Furthermore, a possible correlation between RSV generated from Google Trends and KBV real data regarding the number of cataract surgery and corneal transplantation performed in the period from January 2017 to June 2019 has been analyzed. As well as a possible correlation between the trend search RSV generated from Google Trends and the data of Statista.de regarding the number of performed laser refractive procedures performed from January 2010 to December 2020 (Compare Fig. 1 for study design). To be able to prove or disprove our hypothesis, we contrasted the data and adjusted them in time to each other.

For the statistical analysis SPSS 22.0 (IBM Corp.; Armonk, NY, USA) was used. For the analysis of correlation, the bivariate correlation according to Pearson was used. Data from KBV of each year quarter were correlated with mean value of three months periods of the search intensities on Google trends for specific search terms corresponding to cataract surgery and corneal transplantation. The annual data from Statista.de was correlated with the mean values of a 12 months period search on Google Trends for search terms related to Laser refractive surgery.

### Ethics approval

All procedures performed in studies involving human participants were in accordance with the ethical standards of the institutional and/or national research committee and with the 1964 Helsinki declaration and its later amendments or comparable ethical standards. For this study an ethics approval from the Ethics Commission of the University of Wuppertal was acquired (File number MS/AE 220314).

## Results

### Cataract surgery

**Figure 2:**
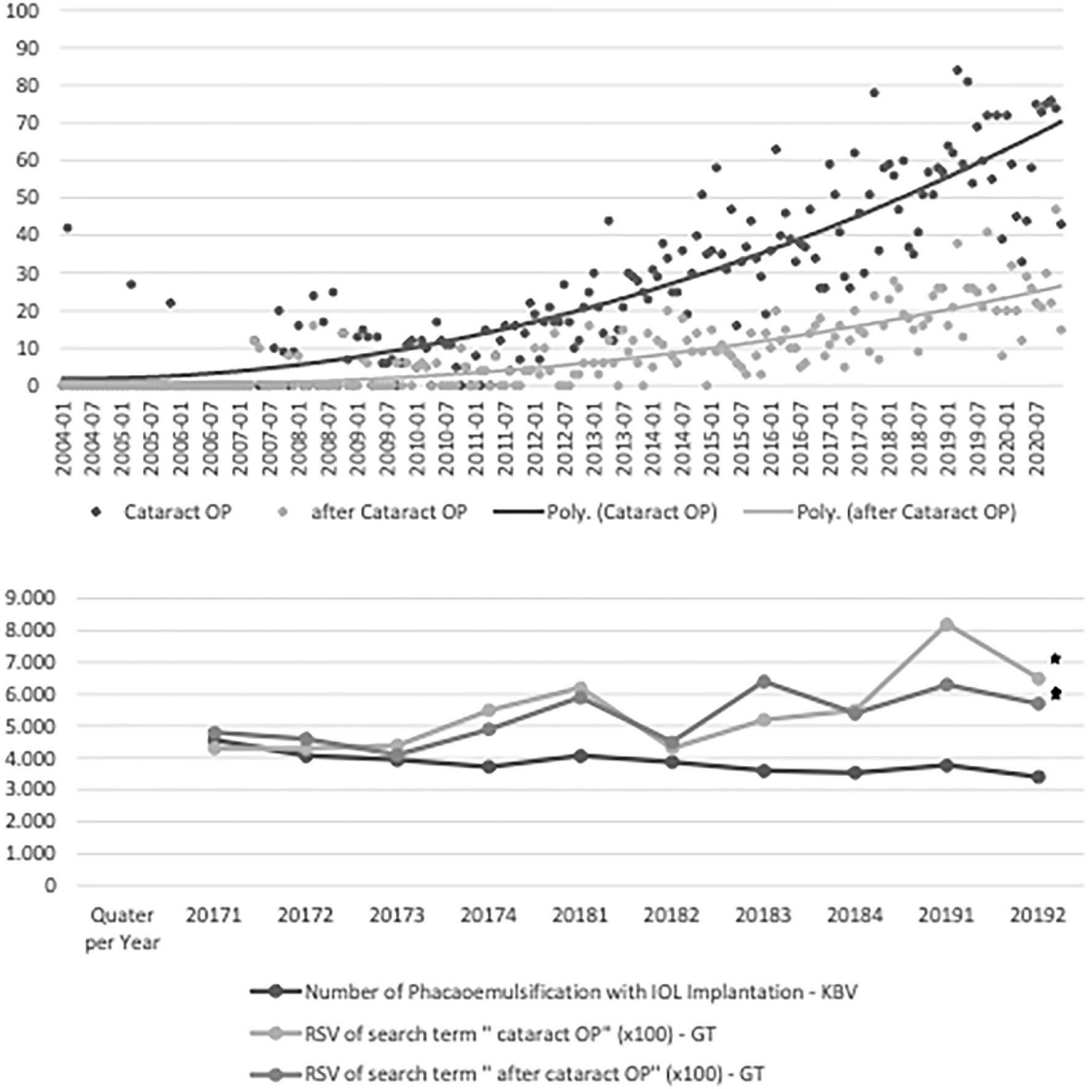
Above) Relative Search Volume (RSV) over time of search terms concerning cataract surgery from 2004 till 2020 in Germany. X-Axis Time (Month/Year), Y-Axis RSV Range 0–100. Below) Correlation between search queries concerning cataract surgery (RSV) and number of cataract surgeries in Germany between March 2017 and June 2019. X-Axis: Time (Year/Quarter), Y-Axis: RSV × 100, Number of cataract surgeries performed. RSV (relative search volume). Star shows statistically significant correlation.

The RSV of search terms related to Cataract surgery such as ‘’Cataract OP- dialect German’’, ‘’after Cataract OP’’ and ‘’Cataract OP’’ increased steadily from 2004 till 2020 (Fig. 2). Other search terms such as “Lens exchange”, “lens operation” and “Lens surgery” have shown no RSV on Google Trends, at all. On the other hand, according to the KBV, the total annual number of cataract surgeries performed increased slightly from January 2017 to June 2019 in Germany. In this context, a statistical correlation between RSV on Google Trends and the number of cataract operations was observed (Fig. 2). Search terms related to cataract surgery have shown a low to moderate correlation between 0.33 and 0.60, depending on the used search term. For exact values of each search term compare Tabel 1.

### Corneal transplantation

The RSV of search terms related to corneal transplantation varied significantly. For example, the trend search for ‘’corneal transplantation’’ remained almost constant in the period from 2004 till 2020 in Germany. On the other hand, the trend search for “DMEK” increased constantly over the same period. Furthermore, since 2014, the “DMEK” trend search increased remarkably and even overlapped the trend search of “corneal transplantation”, which may reflect the increased popularity of DMEK in the last 6 years in Germany. On the other hand, search terms such as “Keratoplasty”, “Donor cornea”, and “lamellar corneal transplantation” have shown low or no RSV on Google Trends.

**Figure 3:**
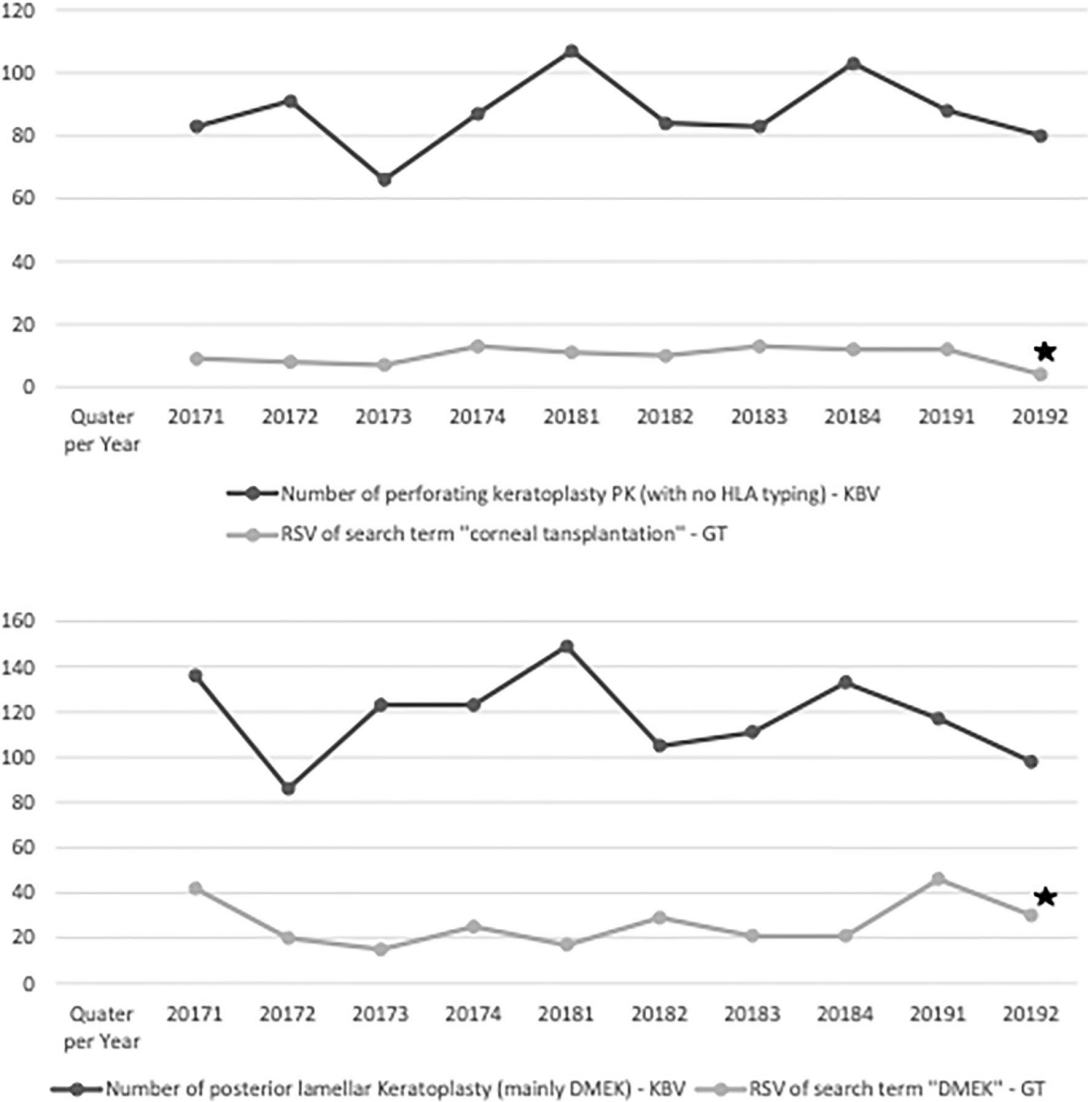
Above) Correlation between the relative search volume (RSV) for “corneal transplantation” and the number of perforating keratoplasty (PK) over time in Germany. X-Axis: Time (Year/Quarter), Y-Axis (RSV, absolute number of PK/quarter). Below) Correlation between the relative search volume (RSV) for “DMEK” and number of DMEK surgeries over time in Germany. X-Axis: Time (Year/Quarter), Y-axis: (RSV, absolute number of DMEK/quarter). Star shows statistically significant correlation.

According to KBV Data, the number of performed PK and DMEK in the period from January 2017 till June 2019 in the out-patient clinic was 872 and 1183, respectively. Furthermore, the number of posterior lamellar keratoplasties (mainly DMEK) increased from 470 cases in 2017 to 615 cases in 2018. Moreover, the number of PK remained almost constant over the same period. Thus, the RSV from Google trends and the performed corneal transplantations could have been analyzed and statistically correlated (Fig. 3). RSV of search terms such as “corneal transplantation” and “DMEK” has shown a significant moderate correlation with 0.69 and 0.552 with *p* < 0.5 respectively in the period from January 2017 to June 2019.

Search terms such as “donor cornea”, “lamellar transplantation” and “keratoplasty” have shown weak correlation with number of performed procedures or even with minimal. In Table 1, RSV and their correlation to the performed surgeries in terms of corneal transplantation have been shown.

**Table 1:** Correlation of relative search volumes (RSV) between specific internet search terms concerning cataract surgery and corneal transplantation in Germany between March 2017 till June 2019 and laser refractive procedures from 2010 till 2020 and totally performed surgical procedures.

German search term (english translation in brackets)	Relative search volume trend	Pearson correlation (2 Tailored)	*p* value
Grauer star OP (cataract surgery)	Increasing	r = 0.59	*p* < 0.05
Nach grauer star OP (after cataract surgery)	Increasing	r = 0.60	*p* < 0.05
Katarakt OP (Cataract surgery)	Increasing	r = 0.33	Not significant
Grauer star OP Erfahrung (Cataract surgery personal experience)	Increasing	r = 0.17	Not significant
Katarakt (cataract)	Constant	r = 0.08	Not significant
Linsen Tausch (Lens Exchange)	No data available	–	–
Katarakt-, Linsen-Chirurgie (Cataract-, Lens- surgery)	No data available	–	–
Linsen operation (lens operation)	No data available	–	–
Hornhauttransplantation (corneal transplantation)	Constant	r = 0.69	*p* < 0.05
DMEK	Increasing	r = 0.55	*p* < 0.05
Keratoplastik (keratoplasty)	Constant	r = 0.23	Not significant
Spenderhornhaut (donor cornea)	No data available	–	–
DALK‚ DSAEK, schichtweise Hornhauttransplantation (DALK, DSAEK, lamellar corneal transplantation)	No data available	–	–
Augenlasern (Eye laser)	Decreasing	r = 0.83	*p* < 0.05
Augen lasern kosten (Costs of eye laser)	Constant	r = 0.69	*p* < 0.05
LASIK	Low	r = 0.18	Not significant
LASIK augen OP (LASIK eye surgery)	Low	r = 0.08	Not significant
PRK, trans PRK	No data available	–	–

DALK = Deep Anterior Lamellar Keratoplasty, DMEK = Descemet Membrane Endothelial Keratoplasty, DSAEK = Descemet Stripping Automated Endothelial Keratoplasty, LASIK = Laser-assisted in-situ Keratomileusis, PRK = Phototherapeutic Keratectomy.

### Laser-assisted corneal refractive surgery

**Figure 4:**
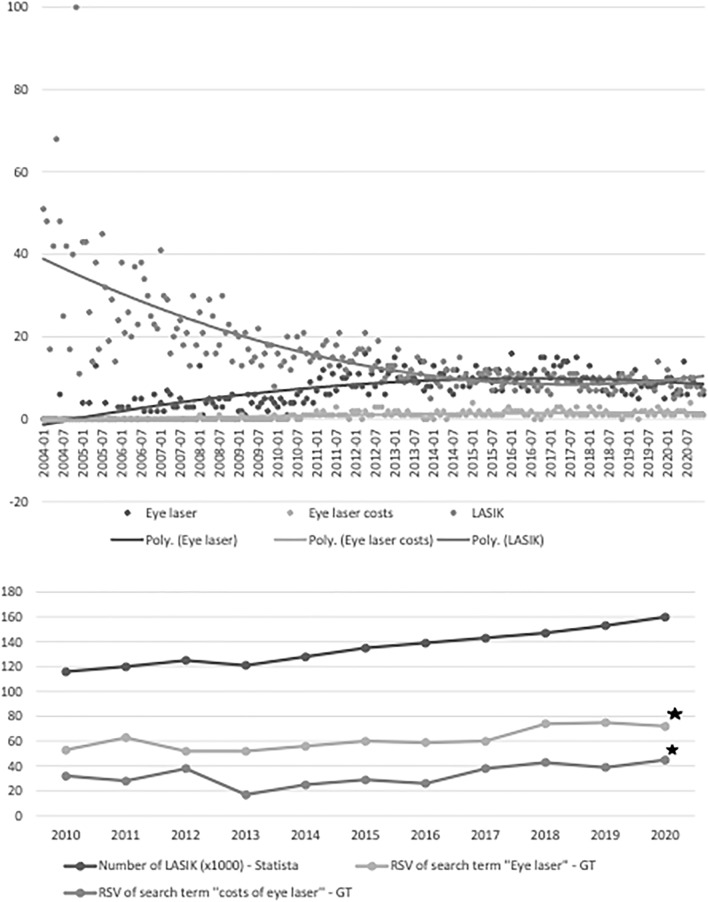
Comparing relative search volumes (RSV) of the search terms ''LASIK'', ''eye laser'' and ''cost of eye laser'' between 2004 and 2020 in Germany. X-Axis: Time (Month/Year), Y-Axis RSV (0–100). Correlation between RSV for “costs of eye laser”, “eye laser” and estimated number of LASIK procedures over time in Germany (Statista.de). X-axis: Time (Year/Quarter), Y-axis: RSV of search term (× 1000), absolute estimated number of LASIK. Star shows statistically significant correlation.

The RSV of search terms related to laser-assisted corneal refractive surgery varied significantly in the period from 2004 till 2020 in Germany. For example, RSV of “LASIK” was highest in 2004, afterwards it decreased steadily till 2013. Since then, RSV remained almost constant. On the other hand, the trend search for “eye laser” and “eye laser costs” increased steadily from 2004 till 2014. Afterwards the trend search remained constant and slightly higher than the RSV of “LASIK” alone (Fig. 4). This might be explained by the increased popularity of other laser-assisted surgical procedures other than LASIK, such as PRK, Transepithelial PRK, Femto-LASIK, and thus the search inquiries were generally for laser-assisted eye surgery rather than specifically for LASIK.

According to Statista.de, the number of laser refractive laser procedures primarily LASIK has been growing steadily over the last 20 years. The number of LASIK increased from 116,000 in 2010 up to 160,000 in 2020 with a factor of 1.3. Moreover, the RSV could have been analyzed and statistically correlated to the number of performed surgeries (Fig. 4). Search terms such as “Eye laser” and “Eye laser costs” have shown moderate to high significant correlation from 0.69 and 0.83 with *p* < 0.05. Further data of the analysis are shown in Table 1.

In terms of PRK and trans-PRK, the RSV was constant since 2004, yet lower than that for LASIK. Despite available data on Google Trends, a statistical analysis was not possible as there were no data regarding the number of procedures done for further correlation.

## Discussion

Overall, the results show that the RSV in Germany on Google Trends for cataract surgery, DMEK surgery, and Laser refractive corneal surgery increased over the last years. On the other hand, the RSV for corneal transplantation as a general search term and specifically for LASIK tend to be constant or even decrease in Germany. Moreover, the RSV on Google Trends has shown a low to high statistical correlation with number of procedures performed in Germany over a specific period of time.

The results presented here allow the following conclusions to be drawn. (1) The RSV for certain corneal cataract and refractive surgery procedures correlates both geographically and temporally with the actual surgeries performed. (2) The correlation is strongly dependent on the search term used for the specific surgical procedure.

With this in mind, future studies should investigate which linguistic terms are actually used by patients. In addition, it is still unclear which age group actually uses search engines on the internet to obtain general information about their disease or an upcoming operation. This could lead to an even higher correlation between the number of operations performed and the actual search volume and thus increase the discriminatory power. Another important limitation is that different age groups and different end device users use divergent platforms (e.g. Twitter, LinkedIn, Facebook, Instagram). This could lead to deviations, as different platforms address different target groups. Nevertheless, GoogleTrends is a particularly attractive platform because it addresses a broad spectrum of age groups and the data is easily accessible.

Nevertheless, the use of certain search terms could also vary depending on patient age and generally over time or even geographically. Also, with a decreasing RSV of, for example, refractive surgery procedures, but at the same time a significant increase in the number of surgeries performed, it is noticeable that hypothetically a general acceptance and dissemination of a surgical procedure leads to patients informing themselves less about it on the internet (e.g., friend-by-friend dissemination) and subsequently posting their experiences more in social media channels. However, access to such data is now severely limited, so unfortunately no statement can be made on this in this study.

Up to now, planning and distribution on a microeconomic level (i.e. the individual hospital) as well as on a macroeconomic level (i.e. the entire health care system) are based only on retrospectively collected case numbers and costs of the past. An adaptation of health strategic questions on the basis of data collected in real time is rare or non-existent so far. However, due to the fast pace of today's world, which is partly caused by a multitude of disruptive processes as well as increased international mobility, a fast adaptation to current developments or technologies is essential to achieve an adequate health economic result. In addition, as the SARS-CoV-19 pandemic currently illustrates, it is advantageous to ensure the flexibility of the health care system and to react in a timely manner to national as well as global developments. For such plasticity in the health care system, it is crucial to be able to collect and evaluate structural health data in real time and, moreover, to be able to react adequately to them. Due to the decentralized structure of the German health care system, but also due to the orientation on data from previous years, it is very difficult in Germany compared to the United Kingdom, for example, to generate data in real time on the geographical and time-dependent occurrence of diseases, the current number of surgical procedures performed or the demand for certain services in the health care system and to be able to react to these at short notice. A data pool of enormous size is generated in real time every day in the form of search engine queries and social media by millions of people in Germany. Alternatives are “online” large registries fed by large proportions of health care providers such as IRIS in the USA (by AAO) or OREGIS (by German Ophthalmological Society; www.oregis.de) in Germany.

The main limitation in validating the above hypothesis is the general lack of availability of official health data. In several countries where data are available, they usually consist of large time interval data (e.g., annual data). Making it difficult to analyze and predict diseases and outbreaks^13^. A similar limitation exists in our study in terms of official health data which is based on each quarter per year, neither real time data nor monthly based ones. On other hand, Despite the fact that the data acquired from google trends i.e. RSV are not absolute data, which in turn difficult to statistically correlate with real data from official authorities, using pearson’s correlation in our study for the data available was an objective statistical methodology to prove i.e. H1 or disprove H0 a correlation between RSV and real data. We assume that further correlation designs can be settled with more available data in the future studies.

In addition, as a limitation, it cannot be clearly distinguished whether the search terms were entered by treating physicians or patients. Furthermore, the search terms used could have been linguistically indirectly specified by the patients' physicians. As the input device in the form of the smartphone is almost ubiquitously available and always linked to the geographical position of the respective input. In the future, real-time acquired data related to eye diseases, treatments and surgical interventions, could be crucial for the immediate and short-term planning and even the strategic orientation of private and public health practices.

## Data Availability

The authors confirm that the data supporting the findings of this study are available within the article. Raw data that support the findings of this study are available from the corresponding author, upon reasonable request.
